# A rare case of pulmonary embolus after arthroscopic meniscus surgery

**DOI:** 10.1093/jscr/rjab101

**Published:** 2021-04-22

**Authors:** Janie Hu, MaKayla Cox, Alexander Yang

**Affiliations:** 1 St. George's University School of Medicine, True Blue, Grenada; 2 Chemistry Department, University of Illinois Springfield, Springfield, IL 62703, USA; 3 Center for Integrative Metabolic and Endocrine Research, Detroit, MI 48202, USA; 4 Center for Molecular Medicine and Genetics, Wayne State University of Medicine, Detroit, MI 48202, USA

**Keywords:** Thromboprophylaxis, Arthroscopic, Meniscectomy

## Abstract

Although there is consensus that thromboprophylaxis is necessary for major orthopedic surgeries such a joint replacement, there is no widespread consensus on the need for thromboprophylaxis for minor arthroscopic surgery. Here, we present a case of deep vein thrombosis (DVT) and pulmonary embolism (PE) after a common arthroscopic meniscectomy in a healthy 20-year-old female collegiate athlete. The patient had no risk factors except for prior use of combined oral contraceptive pills (COCPs). Twenty hours after an uncomplicated right knee meniscectomy, patient presented to ED with right calf pain and cramping, and DVT was confirmed using ultrasound. One week later, patient presented again to ED with dyspnea and chest pain. PE was diagnosed on CT angiography. Despite the rarity of thromboembolic complications in minor arthroscopy surgery, the broadened use of thromboprophylaxis in patients with even few risk factors could prevent thromboembolic complications from occurring.

## INTRODUCTION

The risk of deep vein thrombosis (DVT) resulting in pulmonary embolism (PE), also known as venous thromboembolism (VTE), is known to increase with surgeries that require general anesthesia lasting longer than 30 min. However, even minor surgeries such as knee arthroplasties are known to significantly increase risk of VTE [1]. Even so, the overall incidence of VTE is extremely low and is estimated to be <0.1% [2]. Here, we present a rare case of DVT followed by PE diagnosed by ultrasonography and CT angiography (CTA) after arthroscopic knee meniscectomy in a healthy 20-year-old female collegiate athlete.

## CASE REPORT

Arthroscopic isolated right lateral meniscectomy was performed in a 20-year-old female with MRI confirmed degenerative tear in the posterior horn of the medial meniscus. Patient had a history of four prior left knee meniscectomies and one right knee meniscectomy. Patient was an active collegiate athlete with BMI of 22 with neither vascular disease history nor family history of VTE events. All prior surgeries were done successfully without complications. Two days prior to this surgery, she stopped her use of combined oral contraceptive pills (COCPs). Surgery was completed in 30 min without complications. For routine thromboprophylaxis, patient was encouraged to perform continuous passive motion exercises and given 325 mg aspirin BID for 10 days.

**Figure 1:**
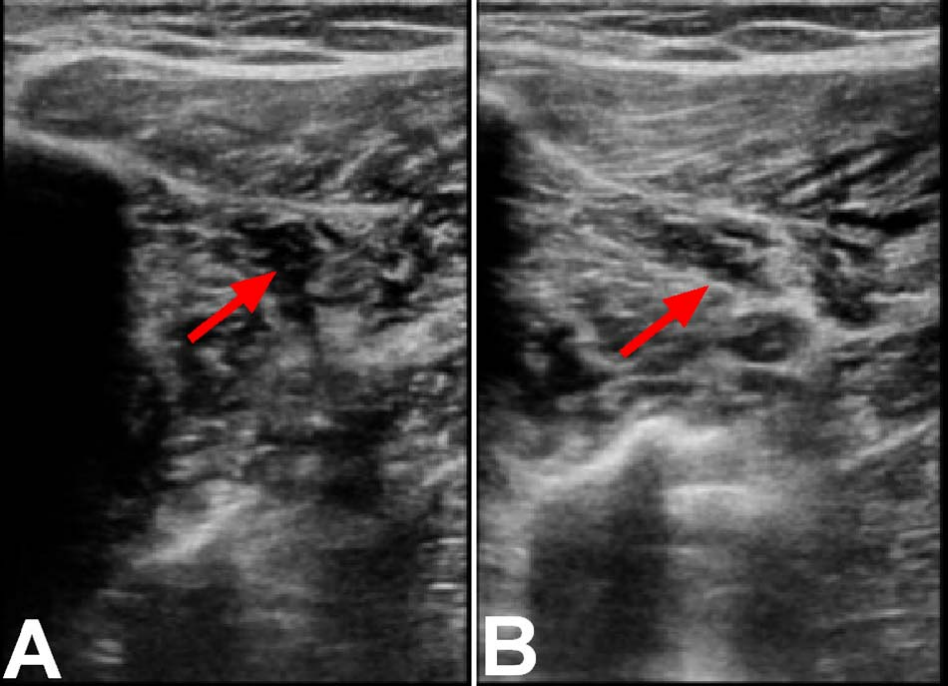
Ultrasound of the right lower extremity within the anterior peroneal vein prior to compression (**A**) and with compression (**B**). Incomplete compression of the vein is suggestive of DVT as indicated by the red arrows.

Twenty hours after discharge from the hospital, patient started to experience right calf pain and cramps. Patient attempted to elevate and ice the right leg as instructed by her surgeon; however, the pain was increasing resulting in admission to the emergency room. Upon presentation to the emergency room, she was able to put pressure on her leg but had difficulty ambulating. Ultrasound of the right lower extremity revealed thrombus within the anterior peroneal vein proximal to the mid-segment that did not extend to the popliteal vein (Fig. 1). Patient was given 4 mg ondansetron and 20 mg apixaban. Upon discharge, patient was given prescription of apixaban with plan of 10 mg BID for a week followed by 5 mg BID for 23 days.

**Figure 2:**
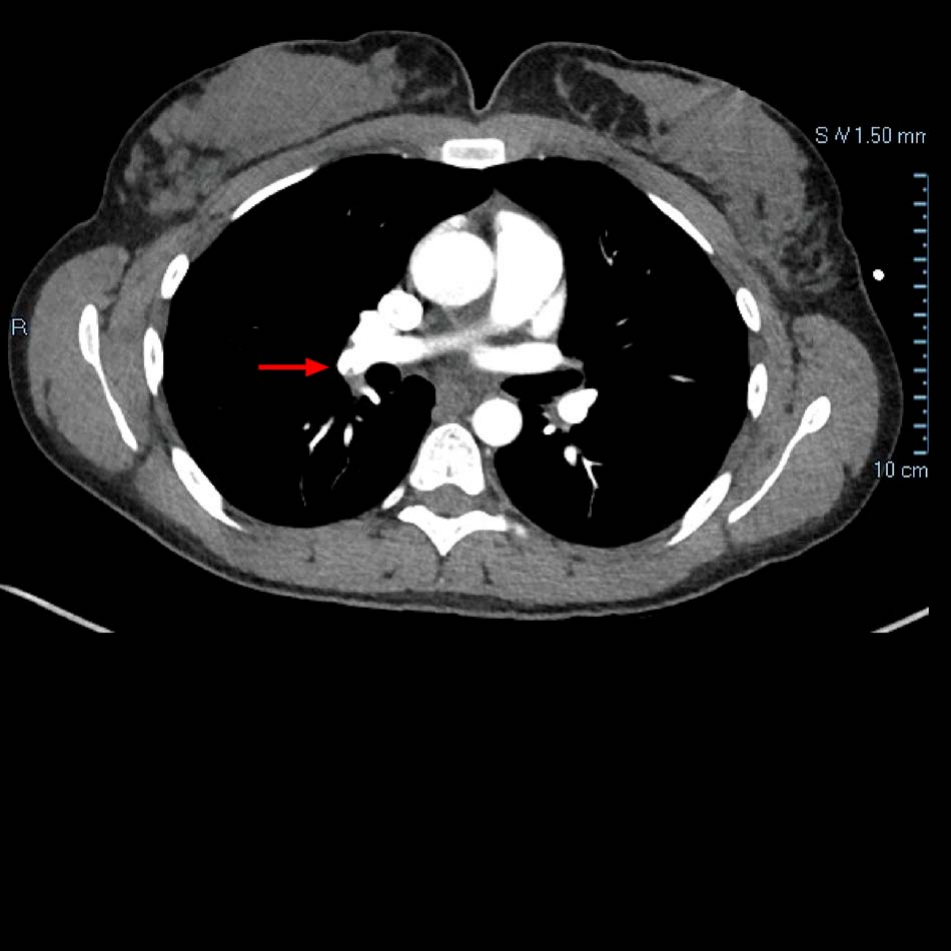
Chest CTA demonstrating filling defect within the right lower lobar pulmonary arterial vasculature consistent with pulmonary embolus as indicated by the red arrow.

One week later, patient presented to ED with dyspnea on exertion and chest pain. Chest CTA demonstrated filling defect within the right lower lobar pulmonary arterial vasculature consistent with pulmonary embolus (Fig. 2). Patient was discharged with plan to follow up with vascular surgery, which continued course of apixaban as previously prescribed.

## DISCUSSION

The risk factors for VTE following surgery are well-defined and include immobility, older age, malignancy, COCPs and thrombophilias [3]. In our patient, use of COCPs was the only identifiable risk factor. The estrogen found in COCPs creates a hypercoagulable state by simultaneously increasing levels of procoagulant factors such as fibrinogen, prothrombin and factors VII, VIII and X, and decreasing anti-thrombin III and tissue factor pathway inhibitor. These effects, albeit dose-dependent, lead to an increased risk of VTE, especially during the initial months of COCP use [4]. COCPs are associated with an increased risk for symptomatic DVT and PE (1.70 and 0.27%, respectively) following arthroscopy of the knee [5].

Although knee arthroscopy is minimally invasive and considered a minor orthopedic surgery, a large population-based case–control study found that knee arthroscopy significantly increases the risk of venous thrombosis, particularly in the presence of acquired or genetic risk factors. In the 3 months post-operation, increased risk of up to 16-fold was found [1]. The risk of thrombosis from knee arthroscopy stems from the use of a tourniquet, which causes stasis of blood flow and hypoxia [1]. Mechanistically, the hypoxia causes damage secondary to inflammation and activation of the coagulation cascade, leading to elevated levels of thrombin–antithrombin complex, plasmin–antiplasmin complex and D-dimer.

While the risk factors and mechanisms of thrombosis are clearly established, there is a lack of consensus of the use of thromboprophylaxis in minor orthopedic surgeries such as arthroscopic knee surgery. A meta-analysis of seven randomized controlled studies on low weight molecular heparin (LMWH) prophylaxis found no significant difference in symptomatic VTE, symptomatic DVT, symptomatic PE and major bleeding between control group and LMWH group [6]. Moreover, there was a significant lower incidence of minor bleeding in the control group compared to the LMWH. As a result, LMWH is not recommended for routine knee arthroscopy in the USA. In Europe, however, prophylaxis with LMWH is recommended in those patients with one or more risk factors.

In an Italian phase II, multicenter, double-blind, placebo controlled randomized control trial, the efficacy of 10 mg rivaroxaban was compared to placebo in preventing mortality, symptomatic VTE and asymptomatic DVT after knee arthroscopy. They found rivaroxaban to significantly reduce the rate of VTE compared to placebo (0.8% vs. 6.1%, respectively, *P* = 0.03) [7]. Furthermore, a recent study found rivaroxaban was associated with 75% lower risk of VTE compared to LMWH (0.2% vs. 1.1%) in an international, parallel-group, randomized, double-blind trial and was not associated with increased risk of bleeding [8]. Taken together, these studies along with our case of VTE in an otherwise healthy 20-year-old female with limited risk factors, advocate for the broadened use of rivaroxaban for pharmacologic thromboprophylaxis in minor orthopedic surgeries.

However, more evidence is needed before universal guidelines can be made. We thus recommend an international multi-center randomized controlled trials (RCTs) testing rivaraxaban as prophylaxis for minor arthroscopic surgery. The results of such a trial would benefit physicians deciding whether thromboprophylaxis is necessary after minor orthopedic surgery.

## AUTHOR’S CONTRIBUTIONS

J.H. and A.Y. organized the text. All authors contributed to the writing of the text.

## CONFLICT OF INTEREST STATEMENT

The authors declare that there are no competing interests.

## FUNDING

This work was supported by the National Institute of Diabetes and Digestive and Kidney Disease F30-DK116529 (A.Y.).
